# Influence of electromagnetic radiation emitted by daily-use electronic devices on the Eyemate® system in-vitro: a feasibility study

**DOI:** 10.1186/s12886-020-01623-6

**Published:** 2020-09-01

**Authors:** Azzurra Invernizzi, Shereif Haykal, Valeria Lo Faro, Vincenzo Pennisi, Lars Choritz

**Affiliations:** 1 Laboratory for Experimental Ophthalmology, University of Groningen, University Medical Center Groningen, P.O.Box 30.001, 9700 Groningen, RB Netherlands; 2 Cognitive Neuroscience Center, Department of Biomedical Sciences of Cells & Systems, University Medical Center Groningen, Groningen, The Netherlands; 3 Department of Ophthalmology, Otto-von-Guericke University Magdeburg, Magdeburg, Germany

**Keywords:** Glaucoma, Intraocular pressure, Eyemate® system, Telemetry, Electromagnetic radiation

## Abstract

**Background:**

Eyemate® is a system for the continual monitoring of intraocular pressure (IOP), composed of an intraocular sensor, and a hand-held reader device. As the eyemate®-IO sensor communicates with the hand-held reader telemetrically, some patients might fear that the electronic devices that they use on a daily basis might somehow interfere with this communication, leading to unreliable measurements of IOP. In this study, we investigated the effect of electromagnetic radiation produced by a number of everyday electronic devices on the measurements made by an eyemate®-IO sensor in-vitro*,* in an artificial and controlled environment.

**Methods:**

The eyemate®-IO sensor was suspended in a sterile 0.9% sodium chloride solution and placed in a water bath at 37 °C. The antenna, connected to a laptop for recording the data, was positioned at a fixed distance of 1 cm from the sensor. Approximately 2 hrs of “quasi-continuous” measurements were recorded for the baseline and for a cordless phone, a smart-phone and a laptop. Repeated measures ANOVA was used to compare any possible differences between the baseline and the tested devices.

**Results:**

For baseline measurements, the sensor maintained a steady-state, resulting in a flat profile at a mean pressure reading of 0.795 ± 0.45 hPa, with no apparent drift. No statistically significant difference (*p* = 0.332) was found between the fluctuations in the baseline and the tested devices (phone: 0.76 ± 0.41 hPa; cordless: 0.787 ± 0.26 hPa; laptop: 0.775 ± 0.39 hPa).

**Conclusion:**

In our in-vitro environment, we found no evidence of signal drifts or fluctuations associated with the tested devices, thus showing a lack of electromagnetic interference with data transmission in the tested frequency ranges.

## Background

Glaucoma is one of the leading causes of irreversible blindness worldwide, with a predicted increase in prevalence as the world’s population continues to age [1]. Furthermore, loss of vision due to glaucoma has been shown to have a detrimental effect on the quality-of-life and mental health of glaucoma patients [2, 3]. While the underlying causes of glaucoma vary, the main controllable risk factor for all subtypes of glaucoma is increased intraocular pressure (IOP) [4].

IOP is usually measured by a trained specialist in a clinical or office setting during working hours. However, IOP exhibits both short and long-term fluctuations throughout a 24-h period [5, 6], which can easily be missed by acquiring static IOP measurements at the clinic in the traditional manner. Although still controversial, some studies have suggested that such fluctuations are an independent risk factor for the development and progression of glaucoma [7, 8]. Therefore, monitoring IOP fluctuations could potentially improve our understanding of glaucoma and how to best control it, and in turn improve patient care. Additionally, glaucoma patients have shown interest in the idea of self-monitoring their condition [9].

Several approaches and devices have been proposed for the continuous monitoring of IOP throughout the day [10]. Currently, the only CE-certified device for continuously monitoring IOP intraocularly is the eyemate® (Implandata Ophthalmic Products GmbH, Hannover, Germany). The eyemate® system comprises a wireless pressure sensor and hand-held device (Mesograph). The sensor communicates with the Mesograph device via electromagnetic coupling, both to provide it with an energy source and to transfer IOP recordings made by the sensor. Implantation of the sensor is usually performed during cataract surgery, where the sensor is placed in the ciliary sulcus after capsular implantation of the intraocular lens. Once implanted, the sensor is meant to remain in the patient’s eye indefinitely [11].

The eyemate® system enables glaucoma patients to measure their own IOP at any time during the day without the need for a doctor’s visit. It also allows ophthalmologists to produce IOP profiles for their patients throughout the day, enabling the detection of any possible fluctuations. Given the novelty of the device, clinical studies of its long-term outcome are still scarce. A study of the long-term safety of the implanted first-generation sensor in 5 open-angle glaucoma patients over an average period of 37.5 months has reported “good functionality and tolerability” [12, 13] . A more recent study of patients who received the implant following Boston Keratoprosthesis surgery reported that the sensor successfully detected postoperative IOP peaks and that measurements made by the sensor showed satisfactory agreement with finger palpation [10].

As the eyemate®-IO sensor communicates with the hand-held reader telemetrically, some patients might fear that the electronic devices that they use on a daily basis might somehow interfere with this communication, leading to unreliable measurements of IOP. In this study, we investigated the possible effect of electromagnetic radiation produced by a number of everyday electronic devices on the measurements made by an eyemate®-IO sensor in-vitro.

## Methods

### Data acquisition

**Fig. 1:**
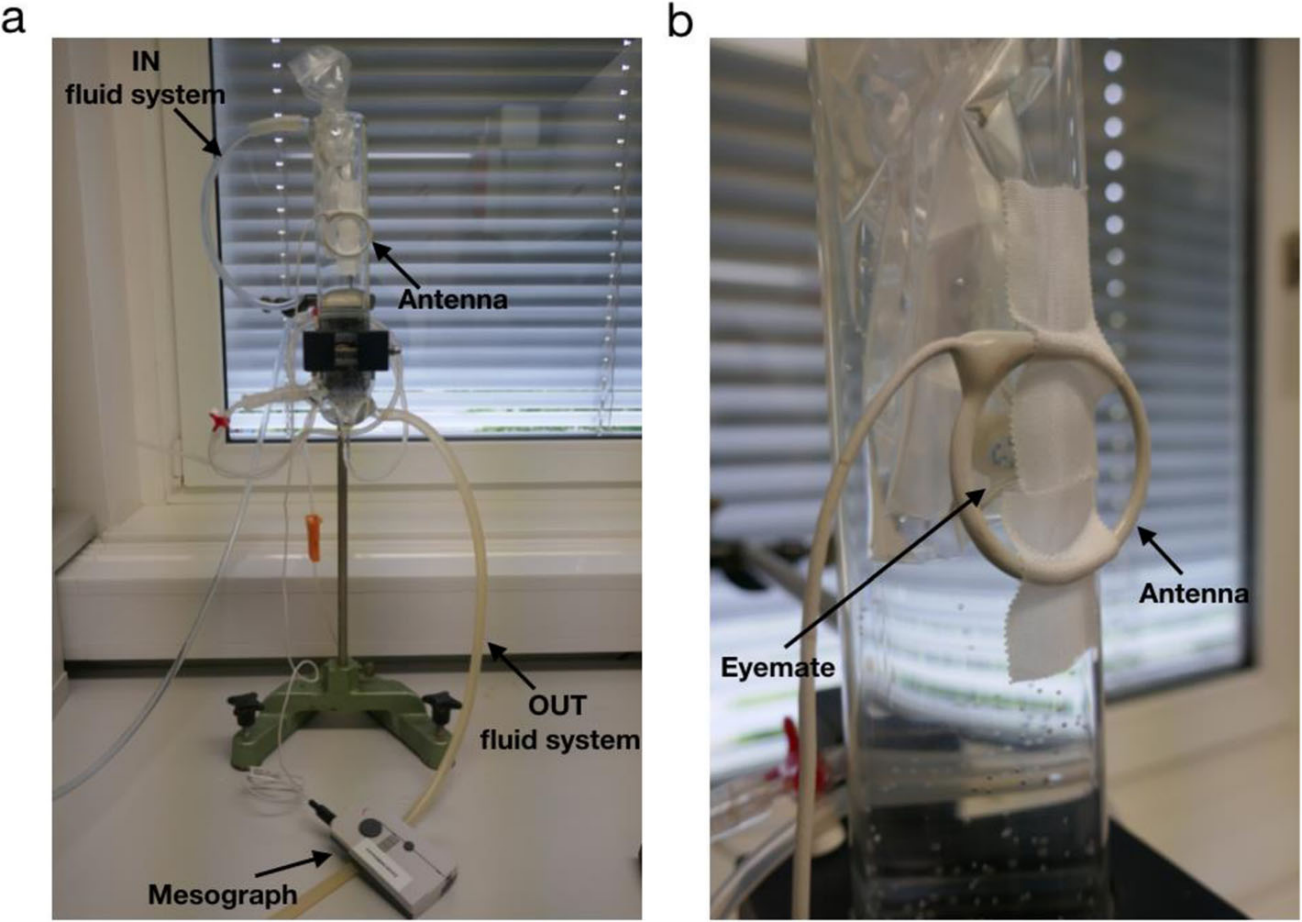
Experimental setup. Panel **a** shows an overview of the setup, including the tissue-bath reservoir, and the Mesograph reader for recording measurements. Panel **b** shows a closer look at the eyemate®-IO sensor and the fixed antenna

The second generation of eyemate® wireless intraocular transducer sensor was used for studying the probable influence of electromagnetic radiation on the IOP measurements. In order to mimic the fixed nature of an implanted sensor, and to ensure a constant distance from the antenna, we suspended the sensor in a plastic bag throughout data collection (Fig. 1, panel b). The sensor was then immersed in a sterile 0.9% sodium chloride solution and placed inside a tissue bath reservoir (RES-01, Experimetria Ltd., Hungary) containing Milli Q-water. Although the system has a temperature sensor to account for temperature changes in the surrounding, we controlled the temperature in our in-vitro environment to closely mimic the stable temperature in the intraocular environment. To do so, we used a circulating water bath (CWB-02, Experimetria Ltd., Hungary) connected to the tissue bath to maintain the temperature constant around 37 °C in the system (Fig. 1, panel a).

An antenna, connected to the Mesograph device, which in turn was connected to a laptop for recording the data, was positioned at a fixed distance of 1 cm from the sensor (Fig. 1, panel b). Approximately two hours (116 min) of “quasi-continuous” measurements were recorded for the baseline and for each device at a sample rate of approximately 10 Hz. To obtain the baseline measurements, any disturbing electromagnetic impulses were eliminated. All plugs were removed from the sockets in the test room, no lights or telephones (fixed line, cordless, or smartphone) were switched on. Only the data acquisition computer and the water pump, placed at 2 m from the experimental setup, were left in the room. The duration of the data acquisition was based on the maximal storing capacity of the readout file (64 KB limit).

In order to have comparable measurements for testing the influence of each electronic device, the same environment was recreated: the plugs were removed from the socket, no lights were switched on and only the data acquisition computer and the water pump were left in the room.

The devices tested were a smartphone (Huawei P10 Lite; radiofrequency range ~ 450–2700 MHz), a cordless phone (Philips D180 Digital Enhanced Cordless Telecommunications (DECT) handset; radiofrequency range 1880–1900 MHz) and a laptop (ASUS ZenBook UX410; radiofrequency range for Bluetooth ~ 2.4 GHz, Wifi ~ 5.15–5.725 GHz). For each device, the experiment was divided into four different measurement intervals: the initial twenty-five minutes were used as “baseline” (hereafter named “no device” to avoid confusion); after this time, each single device was positioned next to the sensor in inactive mode. For the smartphone and laptop, inactive mode constituted setting the device in flight mode, while for the cordless phone it meant putting it in stand-by (no call). The measurements in inactive mode were made for twenty-five minutes, after which the device was switched to active mode for the following twenty-five minutes. This consisted of an active call for the smartphone and the cordless phone, and active Wi-Fi and video streaming for the laptop. The final measurement period consisted of recording measurements with no device for the remaining forty-one minutes.

### Data analysis

**Fig. 2:**
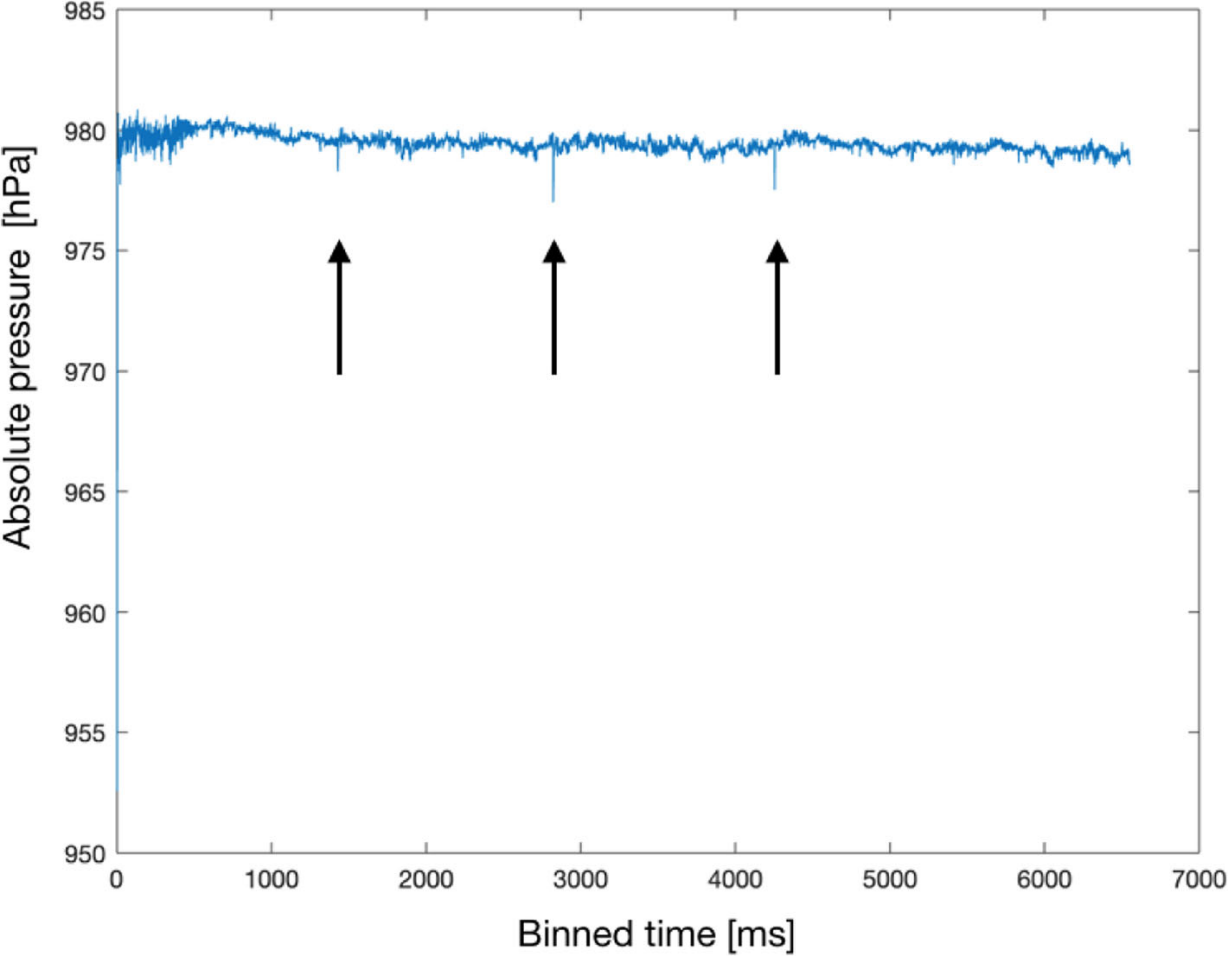
“Quasi-continuous” data recorded for a device. We show an example of the data recorded for one of the three tested devices, namely the smartphone. Arrows indicate drop in the signal measurements

**Fig. 3:**
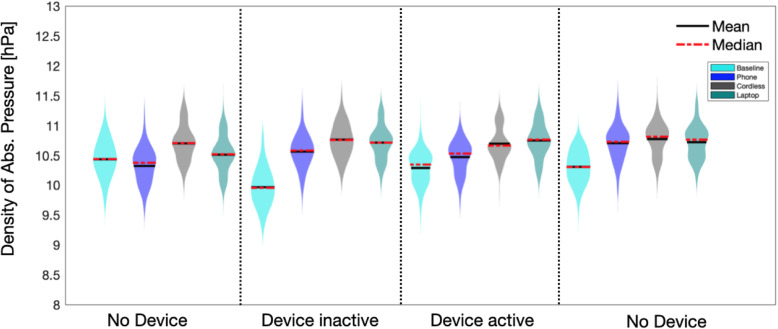
Absolute pressure distributions during the four time-events. A kernel density function was applied to the data for plotting purposes. Mean and median of each distribution are indicated with black solid line and red dotted line, respectively

In order to compare the baseline data with data acquired with different devices, the baseline timeline was divided into four sub-phases corresponding to the four measurement intervals (no device, device inactive, device active and no device) acquired for the devices. Absolute pressure values obtained by the sensor were binned by averaging of ten samples per second (Fig. 2). Using the binned data, we computed the mean, the maximum and minimum values for each measurement period/interval of baseline and devices data. For visualization purposes, a kernel density function was applied to the data to visually evaluate the absolute pressure distribution of each time-event (Fig. 3). The range of fluctuations for each of the four time-events was then determined by calculating the difference between the maximum and the minimum values per data-bin, then by averaging the max and min values for each event. Repeated measures ANOVA with Greenhouse-Geisser correction using SPSS version 25.0 (SPSS Inc., Chicago, Illinois, USA) was used to statistically compare any possible differences between the baseline and the tested devices to investigate the influence of daily use devices on the sensor recording.

The dataset used and analysed during the current study are available from the corresponding author on reasonable request.

## Results

For baseline measurements, the sensor maintained a steady level for the duration of the experiment, resulting in a flat profile with no apparent drift. The same behaviour was observed with the device measurements during active and inactive states.

Small drops in signal measurements were observed corresponding to the time points where each device was handled in the experimental setup (Fig. 2, drops in different time-events are indicated with arrows).

Similar patterns of distribution and ranges of fluctuation were observed for both baseline and devices in all four time-events (Fig. 3 and Table 1). No statistically significant difference (*p* = 0.332) was found between the average fluctuation for each time-events of the baseline and the tested devices.

**Table 1:**
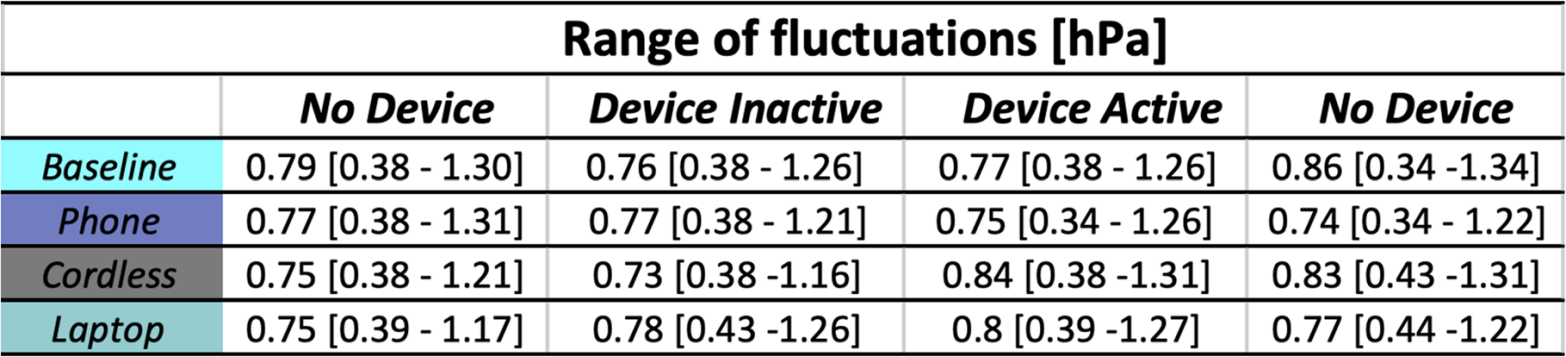
The absolute pressure range fluctuations are reported for the four time-events. For each time-event and each device, we show the averaged and the confidence interval (5 and 95%) of the absolute pressure fluctuations calculated based on the range definition

## Discussion

The eyemate® is a system capable of continuously monitoring IOP intraocularly, designed to be implanted in the patient’s eye during cataract surgery in order to transmit IOP measurements telemetrically [10, 11]. While the sensor is only active when electromagnetically coupled with an external reader device, patients are constantly surrounded by ambient electromagnetic radiation from other electronic devices. Patients might thus fear that their daily use of electronic devices may be a source of interference in the IOP measurements. To date, the number of studies investigating this promising new technology and its potential limitations, such as electromagnetic interference, is still lacking [14–16].

Here, we investigated the interference of electromagnetic radiation emitted by three daily-use electronic devices (a cordless phone, a smartphone and a laptop) on the measurements made by the eyemate® system. We found no evidence of signal drifts or fluctuations associated with the tested devices, indicating a lack of interference of the electromagnetic radiation emitted by the devices on the telemetric transmission of data between the sensor and the antenna of the eyemate® system.

However, abrupt signal drops were revealed in the measurement profiles of the three devices, which corresponded with the time points when each device was handled in the experimental setup. These signal drops are most likely unrelated to electromagnetic interference of the tested device with the sensor, but are probably due to a brief change to the magnetic field emitted by the reader device by moving metallic/conductive components (tested devices) close to the sensor. No reduction in the number of samples recorded was present. Overall, these reported results were to be expected, as electromagnetic coupling is frequency-selective and the communication frequency of the eyemate system was selected to be different from conventional communication frequencies of other wireless electronic devices in order to prevent interference.

A similar study has been previously conducted using the Triggerfish® contact lens sensor (SENSIMED AG, Lausanne, Switzerland) with the same purpose of identifying the influence of electromagnetic radiation on the continuous measurement of the eye pressure by the sensor [14, 15]. The study assessed possible signal drift, noise and fluctuations in IOP measurements recorded by the contact lens sensor due to possible electromagnetic interference from similar daily-use devices. No drift or signal fluctuation was reported.

The Triggerfish® device measures small changes in ocular circumference at the corneal-scleral junction corresponding to changes in intraocular pressure, volume and ocular biomechanical properties as well. While the Triggerfish® contact lens sensor and the eyemate®-IO sensor differ in both anatomical placement and principles of IOP measurement, both sensors share a similar method of telemetric communication with an external antenna for IOP monitoring. Therefore, our current results are in line with those reported for the Triggerfish® [15].

### Limitations

The current study has several limitations. Firstly, the devices were tested a single time each; therefore, reproducibility of our results cannot be claimed and still needs further testing. However, the use of a single sample for each device for a study of this scope is in line with previous work [15]. Secondly, the in-vitro environment we have created is not a perfect replication of the intraocular environment experienced by an implanted sensor. For example, the interaction between external electromagnetic radiation and the organic tissue surrounding an implanted sensor, which might in turn affect the sensor’s readings, was not accounted for. Thirdly, the narrow frequency range of electromagnetic radiation tested limits the applicability of our findings. Future studies may consider testing other daily-use devices, especially those which cover different ranges of electromagnetic radiations.

### Conclusions

Measurements made by the eyemate® system showed no apparent signal drift or evidence of being influenced by external electromagnetic radiation produced by the devices that we tested in our in-vitro environment. Further research using a wider frequency range of electromagnetic radiation is needed to confirm our findings.

## Supplementary information

**Additional file 1.**

**Additional file 2.**

**Additional file 3.**

**Additional file 4.**

## Data Availability

The datasets analyzed in the present study are available in the supplementary material (see Additional Files 1, 2, 3 and 4).

## Abbreviations

ANOVA: Analysis of variance
DECT: Digital Enhanced Cordless Telecommunications
hPa: Hectopascal
IOP: Intraocular pressure
ms: Millisecond
